# No radiographic index predicts difficult intubation using the Optiscope™ in cervical spine surgery patients: a retrospective study

**DOI:** 10.1186/s12871-020-00966-3

**Published:** 2020-02-26

**Authors:** Hyongmin Oh, Hansol Kim, Hyun-Kyu Yoon, Hyung-Chul Lee, Hee-Pyoung Park

**Affiliations:** Department of Anesthesiology and Pain Medicine, Seoul National University Hospital, Seoul National University College of Medicine, 101, Daehak-ro, Jongno-gu, Seoul, 03080 South Korea

**Keywords:** Optiscope™, Videostylet, Difficult intubation, Predictor, Cervical spine surgery

## Abstract

**Background:**

The Optiscope™ can be used for intubation with minimal neck motion. We retrospectively investigated radiographic predictors of difficult intubation using the Optiscope™ by analyzing preoperative radiographic images.

**Methods:**

One hundred eighty-four patients who were intubated with the Optiscope™ under manual in-line cervical stabilization for cervical spine surgery were enrolled. Radiographic indices were measured on preoperative cervical spine lateral X-ray and magnetic resonance imaging images. Difficult intubation was defined as failure or time consumption more than 90 s on the first attempt. To identify significant predictors of difficult intubation using the Optiscope™ and evaluate their diagnostic value, multivariable logistic regression and receiver operating characteristic analyses were used.

**Results:**

Fourty-seven patients showed difficult intubation. There was no significant difference in radiographic indices between the difficult and easy intubation groups, but higher body mass index (BMI) (26.5 [3.0] vs. 24.6 [3.5] kg/m^2^, *P* = 0.001), shorter sternomental distance (SMD) (122.0 [104.0 to 150.0] vs. 150.0 [130.0 to 170.0] mm, *P* = 0.001), shorter interincisor gap (40.0 [35.0 to 45.0] vs. 43.0 [40.0 to 50.0] mm, *P* = 0.006), and higher incidence of excessive oral secretions (10.6% vs. 2.9%, *P* = 0.049) were observed in patients with difficult intubation. In multivariable analysis, BMI (odds ratio [95% confidence interval]; 1.15 [1.03 to 1.28], *P* = 0.011) and SMD (odds ratio [95% confidence interval]; 0.98 [0.97 to 1.00], *P* = 0.008) were associated with difficult intubation with the Optiscope™. In receiver operating characterstic analysis, the area under the curve for body mass index was 0.68 (95% confidence interval; 0.60 to 0.77, *P* < 0.001) and that for sternomental distance was 0.66 (95% confience interval; 0.57 to 0.75, *P* = 0.001).

**Conclusions:**

The incidence of difficult intubation using the Optiscope™ under manual in-line cervical stabilization was 25.5% in cervical spine surgery patients. No significant predictor of difficult intubation with the Optiscope™ was identified among the measured radiographic indices. Although high BMI and short SMD were predictive of difficult intubation with the Optiscope™, their discrimination power was weak.

## Introduction

In patients undergoing cervical spine surgery, endotracheal intubation with direct laryngoscopy is challenging. Application of neck collar or manual in-line cervical stabilization during intubation is necessary to prevent secondary neurologic injury due to excessive neck extension [1]. This maneuver hinders mouth opening and neck extension, resulting in difficult laryngoscopy [2, 3]. For this reason, intubation devices such as videolaryngoscopes, lightwands, flexible fiberoptic bronchoscopes, and videostylets are often used instead of direct laryngoscopes to increase the success rate of intubation and minimize neck motion [4–8].

**Fig. 1:**
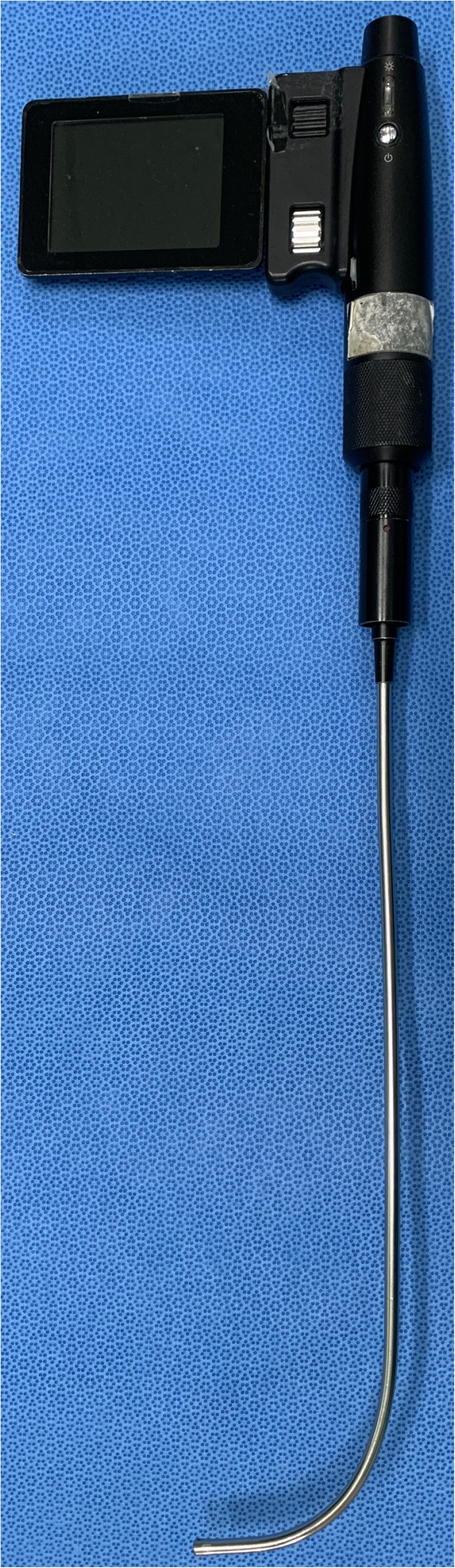
The Optiscope™ used in this study. This videostylet has a rigid stylet that angled 90 degrees,with a camera lens at the bottome end and a handle and monitor at the top end

The Optiscope™ (Clarus Medical LLC, Minneapolis, MN, USA) is a videostylet consisting of a rigid fiberscope with an attached monitor; it is possible to indirectly visualize a patient’s larynx on the monitor during intubation (Fig. 1). Unlike direct laryngoscopes, when intubating with the Optiscope™, alignment of the three airway axes is not necessary. Therefore the Optiscope™ is especially useful in patients with neck motion that must be minimized during intubation. In previous studies comparing the Optiscope™ with other intubation devices, use of the Optiscope™ resulted in less cervical spine motion than was observed with videolaryngoscopes, as well as a shorter intubation time than flexible fiberoptic bronchoscopes, and fewer scooping movements than lightwands [4, 9, 10].

In clinical practice, it is important for anesthesiologists to recognize the factors predicting difficult intubation before anesthetic induction. Numerous studies have shown that body mass index (BMI), Mallampati score, and mouth opening predict difficult intubation with intubation devices such as direct laryngoscopes, videolaryngoscopes, and lightwands [11–14]. Regarding radiographic indices associated with difficult intubation, tongue area, atlanto-occipital gap, mandibulohyoid distance, and the angle of the anterior-inferior point of the upper incisor with the neck in extension are related to difficult laryngoscopy, while epiglottis length is associated with increased intubation time when using lightwands [15–18]. Despite the aforementioned advantages of videostylets, no clinical investigation has yet been performed to identify radiographic predictors of difficult intubation with videostylets.

In this study, we aimed to identify radiographic indices associated with difficult intubation with the Optiscope™ in patients undergoing cervical spinal surgery, by analyzing preoperative cervical spine lateral X-ray and magnetic resonance imaging (MRI) images.

## Methods

### Ethic and approval

Ethical approval for this retrospective study (1909–021-1060) was provided by the Institutional Review Board (101, Daehak-ro, Jongno-gu, Seoul, Korea, 03080) of Seoul National University Hospital (SNUH) on 6 September 2019. The requirement for written informed consent was waived because of the retrospective nature of the study.

### Subject

**Fig. 2:**
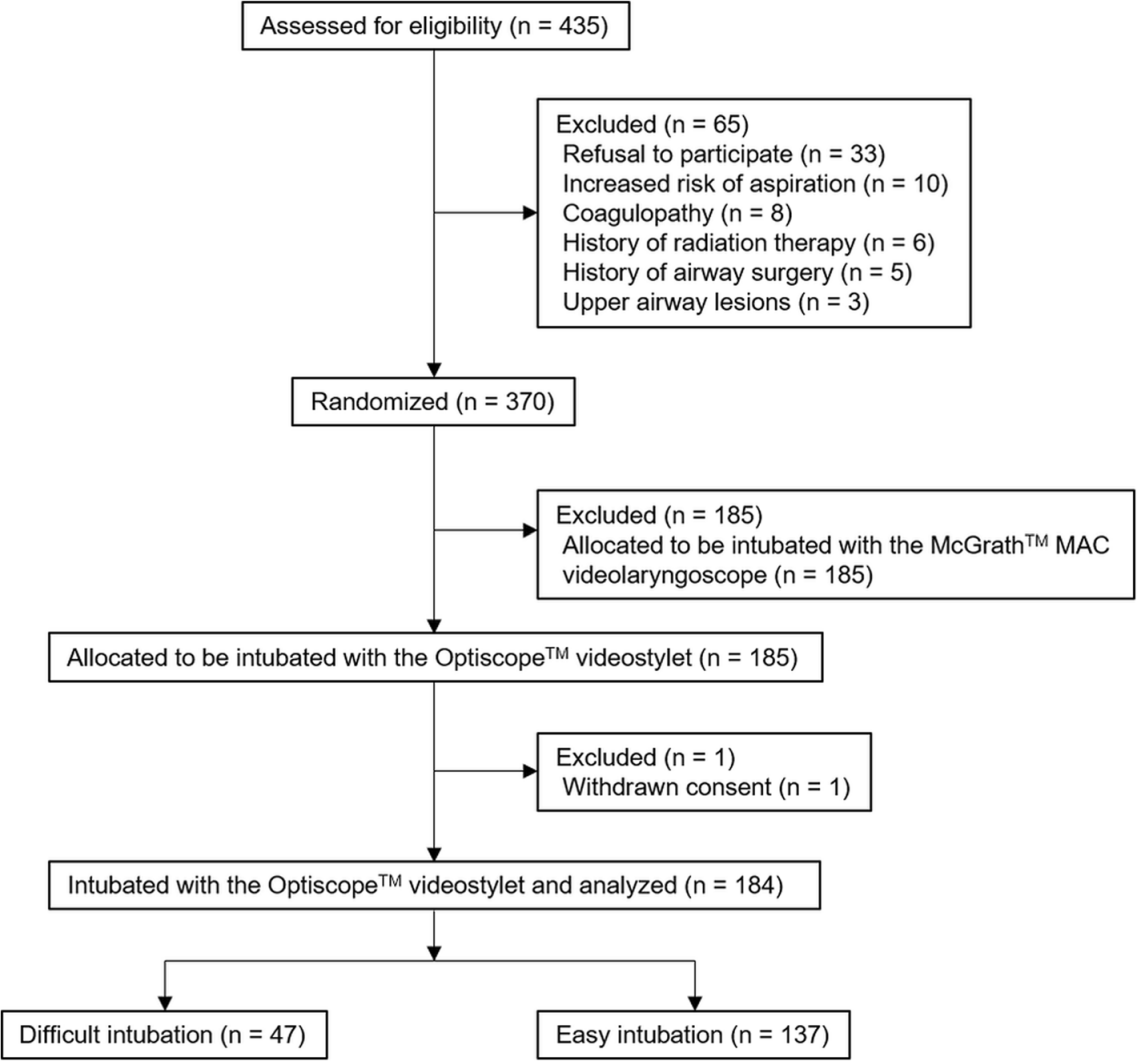
CONSORT flow diagram

Patients who underwent cervical spine surgery and were intubated with the Optiscope™ at SNUH from June 2016 to August 2018 were included (Fig. 2). The participants were previously enrolled in a randomized controlled trial previously conducted at our institution to compare the clinical performance of the Optiscope™ and the McGrath™ MAC videolaryngoscope (Medtronic, Minneapolis, MN, USA) in patients undergoing cervical spine surgery [19]. Patients who were intubated with other intubation devices, and those who had any missing radiographic data, were excluded. Based on the number of intubation attempts and time required for intubation, patients were assigned to either the easy or difficult intubation group. Difficult intubation (the primary outcome measure) was defined as failed intubation or intubation requiring more than 90 s on the first attempt [10].

### Data collection

**Fig. 3:**
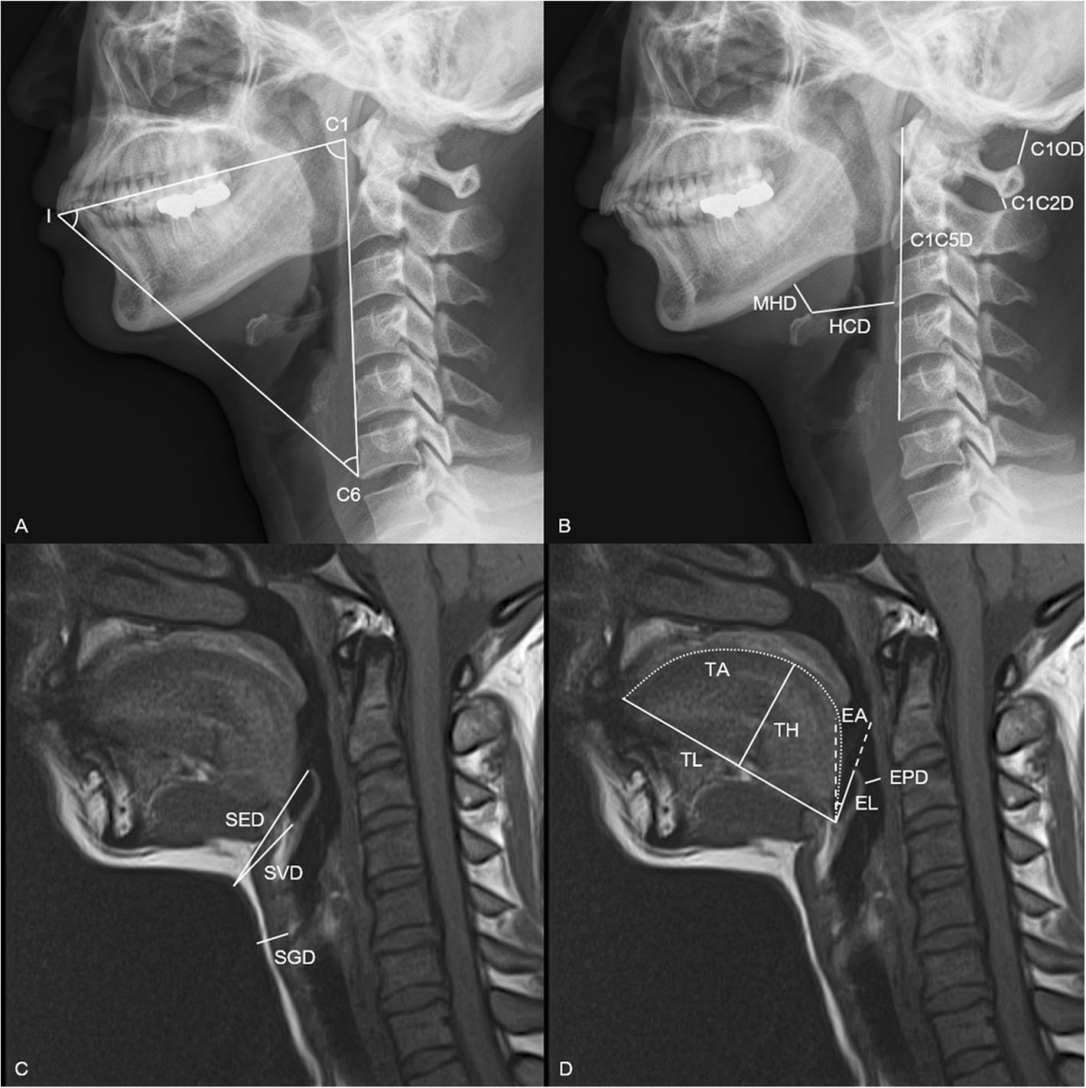
Measurements of radiographic indices investigated in this study. Radiographic indices were measured on cervical spine lateral X-ray (**a** and **b**) and magnetic resonance imaging (**c** and **d**) images in neutral neck position. I. incisor; C1, atlas; C5, the 5th cervical spine; MHD, mandibulohyoid distance; C1C5D, atlanto-the 5th cervical vertebral distance; C1OD, atlanto-occipital distance; HCD, hyoidocervical distance; C1C2D, atlanto-axial distance; SVD, skin-vallecular distance; SED, skin-epiglottic distance; SGD, skin-glottic distance; TL, tongue length; TH, tongue height; TA: tongue area; EL, epiglottis length; EPD, epiglottic-pharyngeal distance, EA, epiglottis angle

General data, including demographic data, American Society of Anesthesiologists physical status, comorbidities, diagnosis, and cervical level operation site were collected. Airway-related variables including the Mallampati score, retrognathia, sternomental distance (SMD), thyromental distance (TMD), and interincisor gap (IIG) were also collected. Twenty-one radiographic indices thought to be associated with difficult intubation with the Optiscope™ were measured three times on preoperative cervical spine lateral X-ray and MRI images, and averaged for analysis by an investigator who was blinded to the group assignments. The radiographic data are shown in Fig. 3 and Table 1. When taking cervical spine X-ray and MRI images, the body and head postions were protocolized in our hospital. In brief, cervical spine lateral X-ray was taken in the standing position with the neutral neck position while MRI images were taken in the supine position with the neutral neck position. When cervical lateral x-ray was taken in the neck extension position, patients were asked to extend the neck without pain or neurologic signs as much as they can. To address potential sources of bias, events that could interfere with intubation with Optiscope™, such as the presence of excessive oral secretions and loose incisor, were also recorded.

**Table 1:** Definitions of radiographic indices investigated in this study

	Detailed description	Meaning
Cervical spine lateral X-ray		
MHD (mm)	Linear distance from the inferior border of the mandibular body to the highest point of the hyoid bone	Tongue size
C1C5D (mm)	Linear distance from the antero-superior border of atlas to the antero-inferior border of the fifth cervical vertebra	Neck length
C1OD (mm)	Linear distance from upper margin of posterior tubercle of atlas to occiput	Neck extension
HCD (mm)	Linear distance from the highest point of the hyoid bone to the anterior border of the nearest cervical vertebra	Tongue size
C1C2D (mm)	Linear distance from lower margin of the spinous processes of atlas to upper margin of the spinous processes of axis in the neutral position	Neck extension
C1-I-C6 (^0^)	The angle between the line from the anterior border of atlas to the tip of upper incisors and the line from the antero-inferior border of C6 vertebral body to the tip of upper incisors in the neutral position	Cervical range of motion
I-C6-C1 (^0^)	The Angle between the line from the tip of upper incisors to the antero-inferior border of C6 vertebral body and the line from the anterior border of atlas to the antero-inferior border of C6 vertebral body in the neutral position	Cervical range of motion
I-C1-C6 (^0^)	The Angle between the line from the tip of upper incisors to the anterior border of atlas and the line from the antero-inferior border of C6 vertebral body to the anterior border of atlas.	Cervical range of motion
C1-I-C6′ (^0^)	Same as C1-I-C6 in the extension position of the cervical spine	Cervical range of motion
I-C6-C1′ (^0^)	Same as I-C6-C1 in the extension position of the cervical spine	Cervical range of motion
I-C1-C6′ (^0^)	Same as I-C1-C6 in the extension position of the cervical spine	Cervical range of motion
Cervical spine MRI		
TL (mm)	Linear distance from the vallecula to the tip of the tongue	Tongue size
TH (mm)	Perpendicular height from the line of tongue length to the top of the tongue	Tongue size
TA (mm^2^)	Tongue area above the line of tongue length from the tip of the upper incisors to the vallecula in the mid-sagittal plane	Tongue size
EL (mm)	Linear distance from the vallecular to the tip of the epiglottis	Epiglottis size
EPD (mm)	Distance between the epiglottis and the posterior wall of the pharynx	Pharyngeal space
EA (^0^)	Angle of epiglottis from perpendicular line	Epiglottis angle
CVLVC	Anatomical position of the vocal cords in relation to the cervical vertebrae	Anatomical position of vocal cord
SVD (mm)	Linear distance from skin to the vallecula	Pre-epiglottic area
SED (mm)	Linear distance from skin to the tip of the epiglottis	Pre-epiglottic area
SGD (mm)	Linear distance from skin to the anterior tip of vocal cords	Pre-cord area

*MHD* Mandibulohyoid distance, *C1C5D* Atlanto-the 5th cervical vertebral distance, *C1OD* Atlanto-occipital distance, *HCD* Hyoidocervical distance, *C1C2D* Atlanto-axial distance, *C1-I-C6* C1-incisor-C6 angle in the neck neutral position, *I-C6-C1* Incisor-C6-C1 angle in the neck neutral position, *I-C1-C6* Incisor-C1-C6 angle in the neck neutral position, *C1-I-C6′* C1-incisor-C6 angle in the neck extension position, *I-C6-C1*′ Incisor-C6-C1 angle in the neck extension position, *I-C1-C6*′ Incisor-C1-C6 angle in the neck extension position; *MRI* Magnetic resonance imaging, *TL* Tongue length, *TH* Tongue height, *TA* Tongue area, *EL* Epiglottis length, *EPD* Epiglottic-pharyngeal distance, *EA* Epiglottis angle, *CVLVC* Cervical vertebral level of vocal cords, *SVD* Skin-vallecular distance, *SED* Skin-epiglottic distance, *SGD* Skin-glottic distance

### Anesthetic management

All patients entered the operating room without any premedication. Following routine monitoring, including noninvasive blood pressure, electrocardiography, and pulse oximetry, anesthesia was induced by target-controlled infusion of remifentanil (effect site concentration, 4 ng mL^− 1^) and propofol (effect site concentration, 4 μg mL^− 1^). Rocuronium was administered at 0.6 mg kg^− 1^ after loss of consciousness to facilitate endotracheal intubation. At least 120 s after rocuronium administration, intubation was performed with the Optiscope™ by one of two attending anesthesiologists, who had each carried out more than 50 successful intubations with the Optiscope™. To decrease inter-intubator variability, only two skilled attending anesthesiologists participated in intubation with the Optiscope™. A reinforced endotracheal tube (internal diameter = 7.0 mm for females and 7.5 mm for males) was used and manual in-line cervical stabilization was performed by another anesthesiologist during intubation of all patients. The endotracheal tube mounted on the Optiscope™ was inserted along the midline and jaw thrust maneuver was performed if entry into the hypopharynx was difficult. Successful intubation was confirmed by continuous end-tidal carbon dioxide monitoring.

### Statistical analysis

Data are presented as number (percent) for categorical variables, mean ± standard deviation for normally distributed variables, and median [interquartile range] for skewed variables. Categorical variables were compared using the chi-square test or Fisher’s exact test. Student’s t test or the Mann–Whitney U test were used to compare continuous variables based on the normality of the data distribution, as assessed by the Shapiro–Wilk test. To identify predictors of difficult intubation with the Optiscope™, univariable and multivariable logistic regression analyses were conducted. Variables with *P* values lower than 0.1 in univariable analysis were included in the multivariable analysis. Receiver operating characteristic (ROC) analysis was performed to assess the diagnostic value of significant variables in multivariable analysis. The predictive accuracy of significant variables was classified into five grades according to their area under the ROC curve (0.5–0.6; fail, 0.6–0.7; poor, 0.7–0.8; fair, 0.8–0.9; good, 0.9–1.0; excellent) [20]. The optimal cutoff point was set to a value that maximized the Youden index (sensitivity + specificity – 1). Subgroup analyses were conducted by dividing into two groups based on the optimal cutoff points. Two-sided *P* values less than 0.05 were considered statistically significant. All statistical analyses were performed using SPSS statistical software (version 25.0; SPSS Inc., Chicago, IL, USA).

In a previous study, difficult intubation as defined in the present study was observed in 10% of patients who were intubated using the Optiscope™ with cervical spine immobilization [10]. To reproduce the proportion of cases of difficult intubation with the Optiscope™ with a 95% confidence interval (CI) and a margin of error of 0.05, at least 159 patients were required in this study.

## Results

A total of 184 patients who underwent cervical spine surgery from June 2016 to August 2018 were enrolled in this study. Among them, 47 (25.5%) and 137 (74.5%) patients experienced difficult and easy intubation with the Optiscope™, respectively.

As shown in Table 2, there was no significant difference in general characteristics between the difficult and easy intubation groups, except for a higher BMI (26.5 ± 3.0 vs. 24.6 ± 3.5 kg m^− 2^, *P* = 0.001) and a greater incidence of excessive oral secretions [5 (10.6%) vs. 4 (2.9%), *P* = 0.049] in difficult intubation group. Among airway-related variables, the difficult intubation group had a significantly shorter SMD [122.0 (104.0 to 150.0) vs. 150.0 (130.0 to 170.0) mm, *P* = 0.001] and shorter IIG [40.0 (35.0 to 45.0) vs. 43.0 (40.0 to 50.0) mm, *P* = 0.006] compared to the easy intubation group. None of the radiographic indices differed significantly between the two groups (Table 3).

**Table 2:** Comparisons of general characteristics and airway-related variables between the difficult and easy intubation groups

	Difficult (*n* = 47)	Easy (*n* = 137)	*P* value
Male (n)	31 (66.0%)	93 (67.9%)	0.808
Age (yr)	57.8 ± 12.1	54.5 ± 14.0	0.154
Weight (kg)	70.5 ± 11.5	67.0 ± 12.7	0.103
Height (cm)	166.0 (158.5 to 171.0)	164.0 (156.0 to 171.0)	0.253
BMI (kg m^−2^)	26.5 ± 3.0	24.6 (3.5)	0.001
BMI > 25 kg m^− 2^	33 (70.2%)	60 (43.8%)	0.002
BMI > 30 kg m^− 2^	8 (17.0%)	8 (5.8%)	0.019
ASA physical status (n)			0.334
1	18 (38.3%)	60 (43.8%)	
2	23 (48.9%)	66 (48.2%)	
3	6 (12.8%)	11 (6.0%)	
Co-morbidity (n)			
Diabetes	10 (21.3%)	21 (15.3%)	0.347
Hypertension	16 (34.0%)	40 (29.2%)	0.533
Cardiac	1 (2.1%)	6 (4.4%)	0.680
Pulmonary	2 (4.3%)	3 (2.2%)	0.603
Neurologic	4 (8.5%)	6 (4.4%)	0.280
Hepatic	0 (0.0%)	9 (6.6%)	0.114
Renal	1 (2.1%)	4 (2.9%)	1.000
Thyroid	0 (0.0%)	2 (1.5%)	1.000
Rheumatoid arthritis	3 (6.4%)	4 (2.9%)	0.374
Diagnosis (n)			
Degenerative	37 (78.7%)	103 (75.2%)	0.623
Tumor	7 (14.9%)	29 (21.4%)	0.349
Trauma	1 (2.1%)	0 (0.0%)	0.255
Congenital	2 (4.3%)	5 (3.6%)	1.000
Operation site (n)			0.961
Above C2	7 (14.9%)	20 (14.6%)	
Below C3	40 (85.1%)	117 (85.4%)	
Mallampati score (n)			0.084
1	8 (17.0%)	37 (27.0%)	
2	20 (42.6%)	58 (42.3%)	
3	15 (31.9%)	37 (27.0%)	
4	4 (8.5%)	5 (3.6%)	
Retrognathia (n)	2 (4.3%)	1 (0.7%)	0.161
TMD (mm)	80.0 (70.0 to 90.0)	80.0 (70.0 to 90.0)	0.485
RHTMD	20.9 (18.9 to 23.4)	21.0 (18.4 to 23.2)	0.785
SMD (mm)	122.0 (104.0 to 150.0)	150.0 (130.0 to 170.0)	0.001
IIG (mm)	40.0 (35.0 to 45.0)	43.0 (40.0 to 50.0)	0.006
Excessive oral secretions (n)	5 (10.6%)	4 (2.9%)	0.049
Loose upper or lower incisor (n)	0 (0.0%)	3 (2.2%)	0.571

*BMI* Body mass index, *ASA* American society of anesthesiologists, *TMD* Thyromental distance; *RHTMD* Ratio of height to thyromental distance, *SMD* Sternomental distance, *IIG* Interincisor gap

**Table 3:** Comparisons of radiographic indices between the difficult and easy intubation groups

	Difficult (*n* = 47)	Easy (*n* = 137)	*P* value
MHD (mm)	14.5 ± 6.2	16.6 ± 9.5	0.080
C1C5D (mm)	103.8 ± 9.6	104.1 ± 10.1	0.835
C1OD (mm)	7.4 (5.1 to 9.2)	7.4 (5.3 to 9.4)	0.720
HCD (mm)	40.4 (36.3 to 44.3)	39.2 (35.8 to 42.8)	0.196
C1C2D (mm)	5.1 (3.0 to 7.1)	4.9 (3.7 to 6.7)	0.956
C1-I-C6 (^0^)	52.0 (49.0 to 55.1)	52.9 (49.5 to 55.4)	0.296
I-C6-C1 (^0^)	40.8 (38.7 to 45.0)	41.6 (38.7 to 45.1)	0.845
I-C1-C6 (^0^)	86.9 ± 9.2	85.4 ± 8.0	0.293
C1-I-C6′ (^0^)	38.3 (34.8 to 41.2)	37.7 (34.8 to 42.1)	0.866
I-C6-C1′ (^0^)	30.8 (29.2 to 32.5)	30.8 (27.7 to 33.9)	0.582
I-C1-C6′ (^0^)	111.2 (107.2 to 115.6)	111.8 (104.7 to 117.2)	0.775
TL (mm)	69.6 (65.7 to 76.2)	69.8 (64.5 to 74.2)	0.634
TH (mm)	36.9 ± 4.6	37.2 ± 5.3	0.743
TA (cm^2^)	19.4 ± 4.0	19.3 ± 3.3	0.810
EL (mm)	18.5 ± 2.2	18.6 ± 2.3	0.810
EPD (mm)	7.0 (5.3 to 9.1)	6.6 (4.8 to 8.2)	0.205
EA (^0^)	31.1 ± 9.6	34.0 ± 11.4	0.126
CVLVC (n)			0.315
C4 level	6 (12.8%)	14 (10.2%)	
C5 level	38 (80.9%)	107 (78.1%)	
C6 level	3 (6.4%)	16 (11.7%)	
SVD (mm)	30.6 ± 4.9	29.4 ± 5.4	0.200
SED (mm)	49.5 ± 7.4	47.4 ± 6.3	0.059
SGD (mm)	12.2 (10.5 to 14.2)	11.5 (9.5 to 13.3)	0.131

*MHD* Mandibulohyoid distance, *C1C5D* Atlanto-the 5th cervical vertebral distance, *C1OD* Atlanto-occipital distance, *HCD* Hyoidocervical distance, *C1C2D* Atlanto-axial distance, *C1-I-C6* C1-incisor-C6 angle in the neck neutral position, *I-C6-C1* Incisor-C6-C1 angle in the neck neutral position, *I-C1-C6* Incisor-C1-C6 angle in the neck neutral position, *C1-I-C6′* C1-incisor-C6 angle in the neck extension position, *I-C6-C1′* Incisor-C6-C1 angle in the neck extension position, *I-C1-C6′* Incisor-C1-C6 angle in the neck extension position, *MRI* Magnetic resonance imaging, *TL* Tongue length, *TH* Tongue height, *TA* Tongue area, *EL* Epiglottis length; *EPD* Epiglottic-pharyngeal distance, *EA* Epiglottis angle, *CVLVC* Cervical vertebral level of vocal cords

The results of multivariable logistic regression analysis are summarized in Table 4. BMI [odds ratio (95% CI); 1.15 (1.03 to 1.28), *P* = 0.011] and SMD [odds ratio (95% CI); 0.98 (0.97 to 1.00), *P* = 0.008] were related to difficult intubation with the Optiscope™.

**Table 4:** Factors for difficult intubation with Optiscope™ on univariable and multivariable logistic regression analyses

	Univariable			Multivariable		
	OR	95% CI	*P* value	OR	95% CI	*P* value
BMI (kg m^−2^)	1.17	1.06 to 1.30	0.003	1.15	1.03 to 1.28	0.011
Excessive oral secretions (n)	3.96	1.02 to 15.42	0.047	4.38	0.88 to 21.90	0.072
IIG (mm)	0.95	0.91 to 0.99	0.024	0.97	0.93 to 1.02	0.238
SMD (mm)	0.98	0.97 to 0.99	0.002	0.98	0.97 to 1.00	0.008
SED (mm)	1.05	1.00 to 1.10	0.064	1.03	0.98 to 1.10	0.257

All variables with *P* < 0.1 in univariable logistic regression analysis were shown in this table and all of them were entered into multivariable logistic regression analysis. Nagelkerke R^2^ statistic was 0.199 and Hosmer and Lemeshow goodness of fit test was not significant at 5% (*P* = 0.814) in multivariable analysis. *OR* Odds ratio, *CI* Confidence interval, *BMI* Body mass index; *IIG* Interincisor gap, *SMD* Sternomental distance, *SED* Skin-epiglottic distance

In ROC analysis, the area under the curve for BMI was 0.68 (95% CI; 0.60 to 0.77, *P* < 0.001) and that for SMD was 0.66 (95% CI; 0.57 to 0.75, *P* = 0.001), both showing poor predictive accuracy. The optimal cutoff points for BMI and SMD were 25.3 kg m^− 2^ and 123.5 mm, respectively. Difficult intubation was observed more frequently in patients whose BMI was higher than 25.3 kg m^− 2^ [odds ratio (95% CI); 3.07 (1.54 to 6.12), *P* = 0.001], or whose SMD was shorter than 123.5 mm [odds ratio (95% CI); 3.89 (1.92 to 7.85), *P* < 0.001].

## Discussion

This clinical study was performed to identify radiographic predictors of difficult intubation with the Optiscope™ in patients undergoing cervical spine surgery with manual in-line cervical stabilization during intubation. Although high BMI and short SMD were associated with difficult intubation using the Optiscope™, no radiographic index measured on preoperative radiographic images predicted difficult intubation with the Optiscope™.

Many radiographic predictors of difficult laryngoscopy have been identified in previous studies. In one such study, a large tongue area measured on preoperative computed tomography was associated with difficult laryngoscopy in acromegaly patients [16]. In another study conducted in patients with cervical spondylosis, a long mandibulohyoid distance and large angle of the anterior-inferior point of the upper incisor in the extended neck position were related to difficult laryngoscopy [17]. A short atlanto-occipital distance has also been reported to make laryngoscopy difficult [15]. However, these radiographic indices did not predict difficult intubation with the Optiscope™ in the current study. This difference may be due to a difference in intubation method between direct laryngoscopes and the Optiscope™. Because alignment of the three airway axes is not necessary when intubating with the Optiscope™, radiographic indices representing neck extension were not predictive of difficult intubation with the Optiscope™. In addition, the Optiscope™ has a slim body compared to direct laryngoscopes. Therefore, when intubating with the Optiscope™, the impact of an enlarged tongue on intubation is less significant.

In this study, BMI was significantly related to difficult intubation with the Optiscope™ based on multivariable analysis. Patients with a BMI higher than 25.3 kg m^− 2^ had a 3.1-fold higher risk of difficult intubation with the Optiscope™. In a previous study investigating the collapsibility index of the upper airway in patients with obstructive sleep apnea, the collapsibility indices in the high and low retroglossal areas were higher in obese versus non-obese patients during sleep, suggesting that obese patients had an increased likelihood of downward movement of the tongue after anesthetic induction [21]. The narrow space between the posterior pharyngeal wall and tongue base can make intubation with the Optiscope™ difficult by hindering its advancement into the hypopharynx. Obesity is known to predict difficult intubation with rigid fiberscopes and lightwands as well as direct laryngoscopes [11]. Although its retromolar or paraglossal aprroach is different from the Optiscope™, the Bonfils™ (Karl Storz Endoscopy, Tuttlingen, Germany), a rigid fiberscope, is similar to the Optiscope™ in terms of its J-shaped structure and scooping movements. In a previous study, the intubation time with the Bonfils™ was longer in patients with small mouth openings, a long TMD, high BMI, and high Cormack and Lehane grade [22]. Lightwand devices also resemble the Optiscope™ in terms of their shape and manipulation type, although they cannot visualize a patient’s larynx during intubation. Previous studies demonstrated that BMI, the Mallampati score, neck circumference, and epiglottis length were positively correlated with intubation time with lightwands [12, 18].

SMD is an indicator of neck length and neck extension. Full extension of the neck makes it easy to align the three airway axes during direct laryngoscopy. A short SMD has thus been identified as a predictor of difficult laryngoscopy [23, 24]. In this study, patients with an SMD shorter than 123.5 mm had a 3.9-fold higher risk of difficult intubation with the Optiscope™. A short SMD can make intubation with the Optiscope™ difficult by impeding its insertion into the oral cavity, due to the hyperacute insertion angle; this increases the chance of lens contamination due to oral secretions. However, in predicting difficult intubation with the Optiscope™, the area under the curve for SMD and BMI was 0.66 and 0.68 respectively. This suggests that their discrimination power is so weak that their role as important predictors of difficult intubation with the Optiscope™ may be clinically insignificant.

Based on our clinical experience, one of the most common difficult situations encountered during intubation with the Optiscope™ is non-visibility of the vocal cord due to the tongue base or epiglottis being in contact with the posterior pharyngeal wall. In several cases, this problem was resolved by the jaw thrust maneuver. Therefore, we expected that radiographic indices related to tongue or epiglottis would be associated with difficult intubation, but that was not the case in this study, possibly due to differences in consciousness and muscle tone at the time of radiographic examination and intubation. In general, the tongue and epiglottis tend to move toward the posterior pharyngeal wall in the supine position in anesthetized patients [25]. We think that the upper airway configuration at the time of intubation may be different from that at the time of radiographic examination.

Until now, there is no consensus definition of difficult intubation using videostylets. In this study, difficult intubation with the Optiscope™ was defined as an intubation duration of more than 90 s or failed intubation on the first attempt. In a previous study comparing clinical performance between the Optiscope™ and Surch-Lite™ lightwand (Aaron Medical, St. Petersburg, FL, USA), [10] an intubation duration of 90 s corresponded to the 95th percentile. Therefore, we set the cutoff point of difficult intubation with the Optiscope™ as 90 s.

This study had several limitations. First, there may have been biases that affected the results due to its retrospective design. Second, there were several cases of difficult intubation with the Optiscope™ due to poor visualization caused by oral secretions. No medication, such as glycopyrrolate, was used routinely before intubation to reduce oral secretions. A previous study reported that glycopyrrolate shortened the intubation time with the Optiscope™, by reducing oral secretions and providing better visualization [26]. Third, since this study was performed in patients who were intubated using the Optiscope™ with manual in-line cervical stabilization for cervical spinal surgery, caution should be taken when applying the results of this study to general patients. In addition, this is a single center study, which also can potentially limit generalizability. Lastly, our predictive model of difficult intubation with the Optiscope™ had relatively weak explanatory power. It is possible that other factors predicting difficult intubation with the Optiscope™ were omitted from the analysis. Further research is needed to identify other significant predictors of difficult intubation with the Optiscope™.

## Conclusion

The incidence of difficult intubation with the Optiscope™ was 25.5% in patients undergoing cervical spine surgery with manual in-line cervical stabilization during intubation. No significant radiographic predictor of difficult intubation with the Optiscope™ was identified on preoperative cervical spine lateral X-ray or MRI images. Although high BMI and short SMD were associated with difficult intubation with the Optiscope™, they had poor predictive accuracy.

## Data Availability

The datasets used and/or analysed during the current study are available from the corresponding author on reasonable request.

## Abbreviations

BMI: Body mass index
C1: Atlas
C1C2D: Atlanto-axial distance
C1C5D: Atlanto-the 5th cervical vertebral distance
C1-I-C6: Atlas-incisor-the 6th cervical vertebra angle in the neck neutral position
C1-I-C6′: Atlas-incisor-the 6th cervical vertebra in the neck extension position
C1OD: Atlanto-occipital distance
C5: The 5th cervical spine
CI: Confidence interval
CVLVC: Cervical vertebral level of vocal cords
EA: Epiglottis angle
EL: Epiglottis length
EPD: Epiglottic-pharyngeal distance
HCD: Hyoidocervical distance
I: Incisor
I-C1-C6: Incisor-atlas-the 6th cervical vertebra angle in the neck neutral position
I-C1-C6′: Incisor-atlas-the 6th cervical vertebra angle in the neck extension position
I-C6-C1: Incisor-the 6th cervical vertebra-atlas angle in the neck neutral position
I-C6-C1′: Incisor-the 6th cervical vertebra-atlas angle in the neck extension position
IIG: Interincisor gap
IRB: Institutional review board
MHD: Mandibulohyoid distance
MRI: Magnetic resonance imaging
ROC: Receiver operating characteristic
SED: Skin-epiglottic distance
SGD: Skin-glottic distance
SMD: Sternomental distance
SNUH: Seoul national university hospital
SVD: Skin-vallecular distance
TA: Tongue area
TH: Tongue height
TL: Tongue length
TMD: Thyromental distance
